# The EEG Split Alpha Peak: Phenomenological Origins and Methodological Aspects of Detection and Evaluation

**DOI:** 10.3389/fnins.2017.00506

**Published:** 2017-09-12

**Authors:** Elzbieta Olejarczyk, Piotr Bogucki, Aleksander Sobieszek

**Affiliations:** ^1^Nalecz Institute of Biocybernetics and Biomedical Engineering, Polish Academy of Sciences Warsaw, Poland; ^2^Department of Neurology and Epileptology, Medical Center for Postgraduate Education Warsaw, Poland

**Keywords:** split EEG alpha peaks, functional brain connectivity, directed transfer function, spectral analysis, average reference, reference electrode standardization technique (REST)

## Abstract

Electroencephalographic (EEG) patterns were analyzed in a group of ambulatory patients who ranged in age and sex using spectral analysis as well as Directed Transfer Function, a method used to evaluate functional brain connectivity. We tested the impact of window size and choice of reference electrode on the identification of two or more peaks with close frequencies in the spectral power distribution, so called “split alpha.” Together with the connectivity analysis, examination of spatiotemporal maps showing the distribution of amplitudes of EEG patterns allowed for better explanation of the mechanisms underlying the generation of split alpha peaks. It was demonstrated that the split alpha spectrum can be generated by two or more independent and interconnected alpha wave generators located in different regions of the cerebral cortex, but not necessarily in the occipital cortex. We also demonstrated the importance of appropriate reference electrode choice during signal recording. In addition, results obtained using the original data were compared with results obtained using re-referenced data, using average reference electrode and reference electrode standardization techniques.

## Introduction

There has been increased interest in understanding the mechanisms of generation of one of the basic patterns of electroencephalographic (EEG) activity—alpha waves. An individual alpha frequency depends on many factors, including age, gender, level of sleepiness, or presence of neurological disorder. All these factors can have an impact on the separation of theta and alpha bands (Klimesch, 1999; Garn et al., 2012; Grandy et al., 2013a; Bazanova and Vernon, 2014). Research shows that alpha power is lower in early childhood and in the elderly than in adulthood (Chiang et al., 2011; Cottone et al., 2013; Grandy et al., 2013b; Ponomareva et al., 2013; Soroko et al., 2014; Vysata et al., 2014; Miskovic et al., 2015; Zappasodi et al., 2015), and that alpha peak frequency is higher in females than in males in posterior parts of the brain (Langrova et al., 2012; Garces et al., 2013). Moreover, alpha power decreases and theta power increases during the transition from wakefulness to sleep (Klimesch, 1999). Change in spectral characteristics of EEG patterns has also been observed in various neurological disorders (Garces et al., 2013; Ponomareva et al., 2013; Zappasodi et al., 2014; Vollebregt et al., 2015).

It is well-known that the amplitude of alpha is higher at occipital relative to frontal derivations, and that alpha peak frequency is higher at occipital relative to frontal electrodes. The frequency difference in alpha peaks between frontal and occipital lobes can result in overlapping double peaks, so called “split alpha.” The split alpha effect was predicted by a model of alpha rhythm generation (Robinson et al., 2001, 2003; O'Connor and Robinson, 2004; Xiong and Yao, 2005; Gray and Robinson, 2013), and was previously observed in a study of healthy volunteers (Chiang et al., 2011). Chiang et al. developed a method for the automatic identification of multiple alpha peaks in EEG data (Chiang et al., 2008).

Robinson et al. (2003) studied a modified model of the corticothalamic system and demonstrated that splitting of the spectral alpha peak can result from spatial brain heterogeneity. Alpha frequency and power are greater in occipital part of the brain compared to the frontal ones. Dominance of the occipital alpha power seems to be driven by a decrease in cortical and an increase in thalamic activity, while the frequency shift may be driven by a decrease in the corticothalamic propagation delay (O'Connor and Robinson, 2004). Spatial heterogeneity may be associated with the diversity of functions associated with lower (8–11 Hz) and higher (11–13 Hz) frequency alpha. Specifically, upper alpha is implicated in cortical processes related to semantic memory, whereas low alpha is implicated in processes related to attention (Klimesch, 1999). Increased upper alpha and decreased lower alpha power have been observed in patients with mild cognitive impairment and Alzheimer's disease, relative to normal elderly subjects (Moretti et al., 2012; Ponomareva et al., 2013).

The aim of this paper was to explain the mechanism of split alpha generation. EEG signals were analyzed using spatiotemporal maps of EEG amplitude and spectral analysis. In addition, Directed Transfer Function (DTF), a method used to evaluate functional brain connectivity, was used to determine the sources of alpha activity with different peak frequencies. We have demonstrated that the mechanism of split alpha generation is much more complicated than was predicted by the Robinson et al. model. In particular, we found that choice of reference electrode and the window size were important factors in the identification of split alpha.

## Materials and methods

### Subjects

EEG was performed in a group of 27 patients who varied in age and sex (23 females, 4 males; mean age: 29.9 ± 11.5 years), and consisted primarily of ambulatory patients with headache, fainting, loss of consciousness, or epilepsy. Patient EEG data were included in analyses if they demonstrated adequate expression of alpha waves.

### EEG registration and preprocessing

EEG data were acquired with the sampling frequency of 250 Hz in a standard 10–20 system of electrode placement using ELMIKO EEG DigiTrack™ Recording System with 19 EEG channels: Fp1, Fp2, F7, F3, Fz, F4, F8, T7, C3, Cz, C4, T8, P7, P3, Pz, P4, P8, O1, O2. Depending on the specified recording conditions, the original EEG signal was recorded using four different reference electrodes: linked earlobes (A1–A2), neck (NK), chin (S1), and frontal (AFz).

Then, the data were re-referenced using average reference electrode (AVERAGE) (Nunez and Srinivasan, 2006) and reference electrode standardization techniques (REST) (Yao, 2001; Zhai and Yao, 2004; Yao et al., 2005). REST is a method that allows for the transformation of original EEG data—with the reference electrode placed at an arbitrary point on the head—to a new dataset with the reference at infinity and the potential at zero or a constant. This transformation was performed using the freely available REST Toolbox (http://www.neuro.uestc.edu.cn/rest/). The procedure is based on the calculation of the leadfield matrix for the canonical concentric-three-spheres head model.

Next, the current source density (CSD) or scalp surface Laplacian was estimated from the transformed EEG data to reduce the impact of volume conduction (Kayser and Tenke, 2006a,b, 2015; Kayser, 2009). These calculations were performed using a spherical spline algorithm (Perrin et al., 1989, 1990; Jurcak et al., 2007) in the CSD Toolbox (http://psychophysiology.cpmc.columbia.edu/Software/CSDtoolbox/).

### Spatiotemporal and spectral analysis

Spatiotemporal maps of EEG patterns were analyzed using the EEG Time-Potential Mapping Module of ELMIKO EEG DigiTrack™ Recording System, which is widely used in clinical practice (Sobieszek, 2009, 2013, 2015).

The power spectrum density (PSD) of EEG signal for each channel was calculated in the range of alpha band from 8 to 13 Hz. Maps of relative spectral power were estimated for different frequency ranges (theta: 4–7 Hz; alpha: 7–8, 8–10, 10–13 Hz; beta: 13–25 Hz).

### Directed transfer function (DTF)

DTF is a measure based on Granger Causality, but is defined in the frequency domain (Kaminski and Blinowska, 1991).

For a multivariate k-channel process, **X**(t) = [X_1_(t), X_2_(t),…, X_k_(t)]^T^, the multivariate autoregressive model takes the form:

(1) $$\begin{array}{l} X(t)=\sum_{m\;=\;1}^{p}\hat{A}(m)\cdot X(t-m)+E(t) \end{array}$$

where **E**(*t*) is a k–dimensional vector, $\hat{A}$ is a square k × k matrix.

We can rewrite (Equation 1) in the form;

(2) $$\begin{array}{l} E(t)=\sum_{m\;=\;0}^{p}\hat{A}(m)\cdot X(t-m)\\ A(0)=I,A(m)=\hat{A}(m)for\;m=1,\ldots ,p \end{array}$$

Transforming the multivariate autoregressive model to the frequency domain, we obtain:

(3) $$\begin{array}{l} E(f)=A(f)X(f),\;whereA(f)=-\sum_{m\;=1}^{k}A(m)\cdot e^{-i\cdot 2\pi \cdot f\cdot m}\\ \;\to X(f)=A^{-1}(f)E(f)=H(f)E(f) \end{array}$$

The matrix of coefficients **H**(*f*) is called the transfer matrix.

The DTF is defined as a normalized version of the transfer matrix:

(4) $$DTF^{2}{\;}_{j\to i}(f)=\frac{|H_{ij}(f){|}^{2}}{\overset{k}{\underset{j=1}{\sum }}|H_{ij}(f){|}^{2}}$$

In the calculation of DTF, the product of the model order and the number of EEG channels must be several times smaller than the number of the samples in the analyzed signal (Blinowska and Kaminski, 2006). In this study, the model order was equal to 10 and the number of EEG channels was 19. The DTF was calculated for 2-, 4-, and 8-s segments sampled with frequency of 250 Hz, and the rule was found to be satisfied.

### Relation between power spectrum and DTF

The power spectrum and DTF have the following relationship (Blinowska et al., 2004):

(5) $$\begin{array}{l} S(f)=X(f)X^{+}(f)=H(f)E(f)E^{+}(f)H^{+}(f)=H(f)VH^{+}(f) \end{array}$$

where **V** = **E**(*f*)**E**^+^(*f*) is the spectral matrix of input white noise processes that does not depend on frequency; + refers to the Hermitean transpose, i.e., the composition of transposition and complex conjugation of a matrix.

The power spectrum, **S**(*f*), depends only on the EEG amplitude. The power spectrum does not depend on the phase of the signal, which gives information about the time relations between signals, and therefore enables the estimation of directionality of the EEG activity propagation. Moreover, the source of EEG activity does not necessarily have to be located at the power spectrum maximum (Kaminski et al., 1997). Thus, the DTF provides additional information to the spectral analysis regarding the localization of generators and the directionality of signal propagation.

The multivariate model used in the DTF calculation already includes all EEG signals and their relations. Thus, the method provides the whole spectral matrix at once, with auto-spectra on the diagonal and cross-spectra on the off-diagonal. The power spectra presented in this paper correspond to the auto-spectra derived from the DTF method.

### Indices based on graph theory

In graph theory, the brain is modeled as a graph composed of nodes, representing brain regions (i.e., the EEG channels, here), and links between the nodes, representing functional connections (i.e., the magnitude and directionality of DTF, here).

For each of the obtained graphs, three indices were calculated: density, degree, and strength (Rubinov and Sporns, 2010). The *degree* of an individual node is equal to the number of links connected to that node, and reflects the relative importance of a node in the network. The mean network degree is commonly used as a measure of the graph *density*, or the total “wiring cost” of the network. The directed variant of the degree distinguishes the number of inward links from the number of outward links, while the weighted variant of the degree, termed the *strength*, is defined as the sum of all neighboring link weights.

## Results

The spectral analysis allows for the identification of split alpha effect, i.e., the presence of two or more peaks with close frequencies in the power spectrum. However, the observed power spectrum depends strongly on choice of reference electrode (Yao et al., 2005), and may therefore influence the split alpha effect. In our data, several patients illustrate the impact of choice of reference electrode, in addition to window size and volume conduction.

### The impact of window size on the identification of split alpha

**Figure 1:**
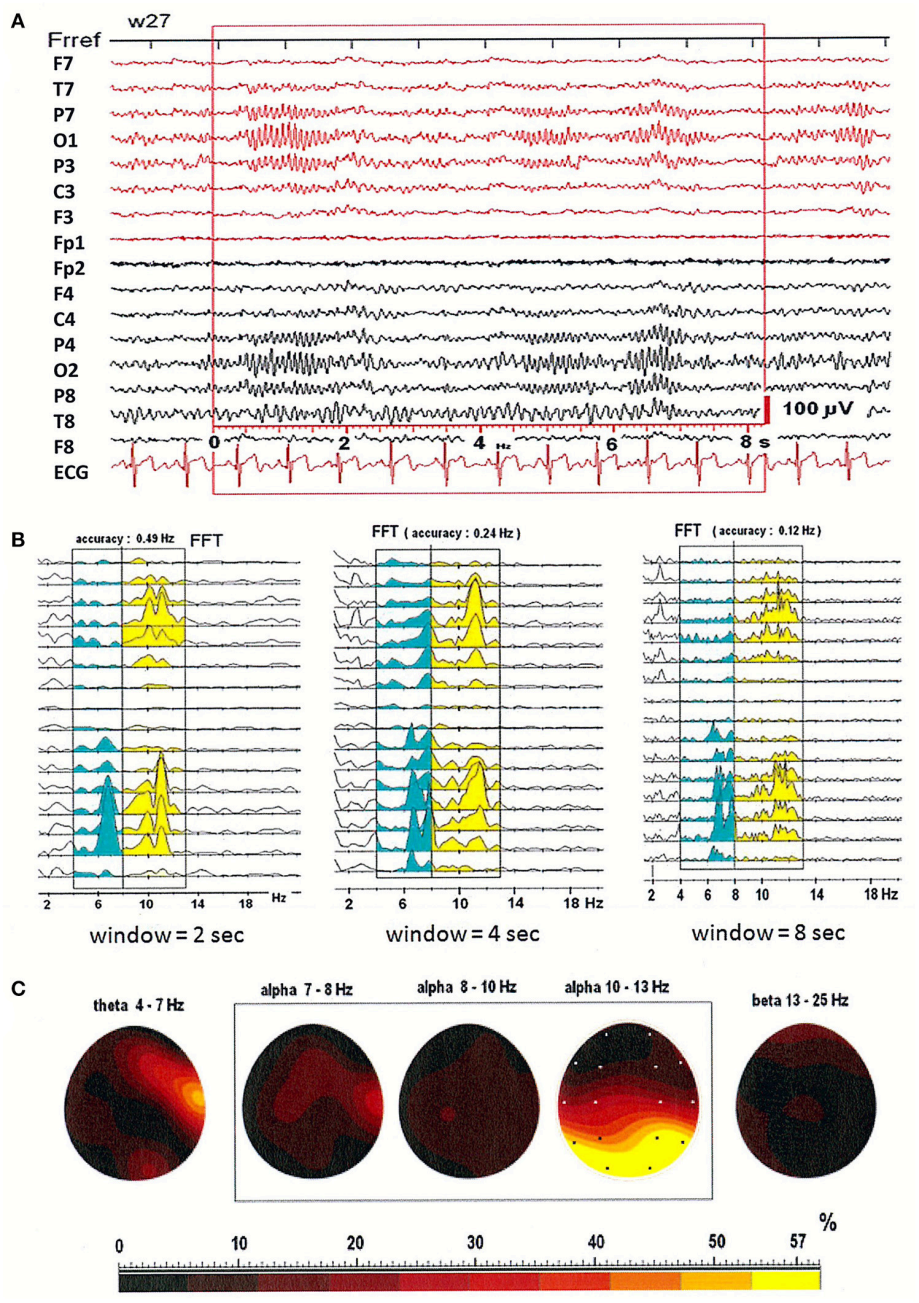
**(A)** EEG recording in the first patient, a 27-year-old woman; **(B)** The power spectral density calculated for 2-, 4-, and 8-s windowed segments of the EEG record; **(C)** The relative power spectra for five frequency bands (theta: 4–7 Hz; alpha: 7–8, 8–10, 10–13 Hz; beta: 13–25 Hz) for a segment of data.

Figures 1A,B illustrates that patterns of spectral power distribution (SPD) depend on choice of window size. EEG was recorded in three window durations: 2-, 4-, and 8-s. Figures 1A,B shows the relative SPD in the first patient, a 27-year-old woman. The split in higher alpha (10–13 Hz) is clearly visible in the shorter, 2-s window. In the longer, 8-s window however, two peaks were observed in the SPD with maximum at electrodes O2 and T4 in theta and low alpha bands, and a broad SPD in the higher alpha range (see Figure 1B). Maps of the relative power spectra are shown separately in five frequency bands (theta: 4–7 Hz; alpha: 7–8, 8–10, 10–13 Hz; beta: 13–25 Hz) in Figure 1C. Increased level of relative power spectrum can be seen clearly at electrodes T4 and O2 in theta (4–7 Hz) and low alpha (7–8 Hz) ranges (areas marked with yellow and red color in Figure 1C). Moreover, high level of relative power spectrum is observed in the posterior part of the brain, with dominance in the right hemisphere at electrode O2 in the higher alpha band (10–13 Hz).

### Comparison of bipolar montage with monopolar montage

**Figure 2:**
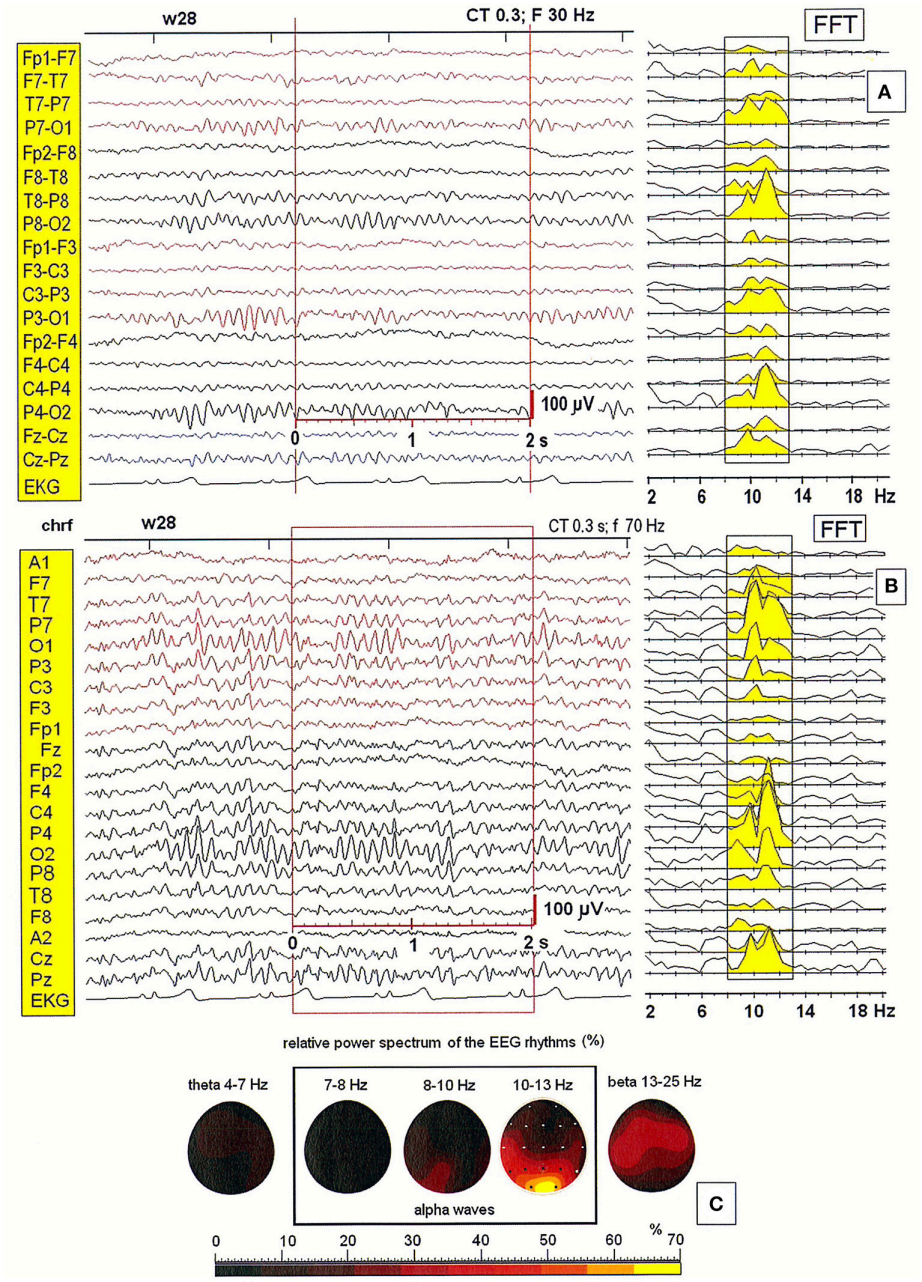
**(A,B)** EEG recording and the power spectral density calculated for the 2-s segment of the EEG record in the second patient (28-year-old woman), recorded using two different montages: bipolar and monopolar (S1); **(C)** The relative power spectra for five frequency bands (theta: 4–7 Hz; alpha: 7–8, 8–10, 10–13 Hz; beta: 13–25 Hz) for a segment of data.

Next, we tested the importance of the location of the reference electrode. Figure 2 shows a comparison of EEG patterns recorded from a second patient (28-year-old woman) using two montages: bipolar (BIP) and monopolar. Four different reference electrodes were evaluated in the examined patients: linked earlobes (A1–A2), neck (nk), chin (S1), and frontal (AFz). Only the monopolar montages allowed for the correct localization of alpha waves' generators. The effect of split alpha is clearly observed in the PSD for both montages. However, comparing the PSD obtained with the bipolar montage (see Figure 2A) with that obtained with the monopolar one (c.f. Figure 2B) revealed the existence of hemispheric asymmetry with dominance of lower frequency alpha in the left hemisphere, and higher frequency alpha in the right hemisphere. The highest relative power spectrum was observed in the posterior part of brain (O1, O2) in higher alpha band (10–13 Hz). A dominance of lower alpha band (8–10 Hz) in the left hemisphere is also evident (see Figure 2C).

### Usefulness of spatiotemporal maps of EEG patterns in daily clinical practice

**Figure 3:**
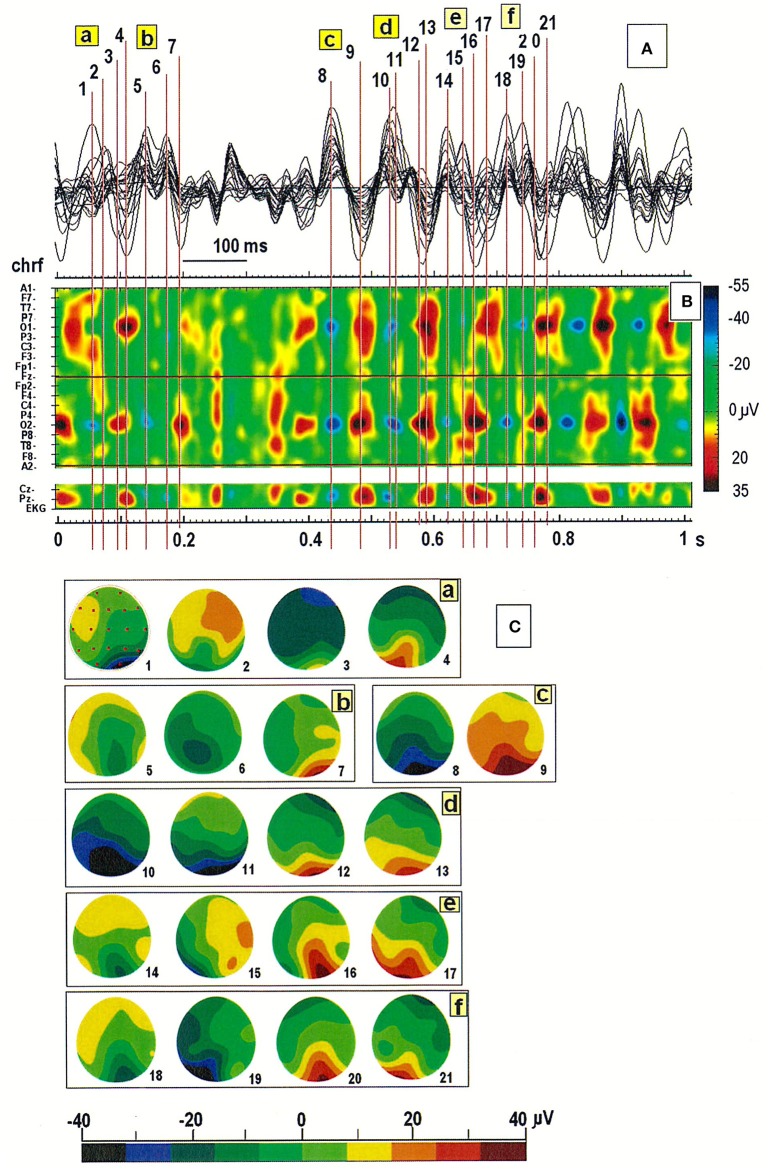
**(A)** Superimposition of a 1-s segment of the EEG record in the same patient as in Figure 2; **(B)** Spatiotemporal map of this segment; **(C)** Six cycles in the EEG recording marked with the letters a-f in **(A,B)**. Individual map corresponds to the relative numbers from 1 to 21 in **(A,B)**.

Inspection of the spatiotemporal map of the EEG pattern in one patient revealed the existence of two sources of alpha waves, localized mainly in the occipital derivations (O1 or O2; see Figure 3). Slightly different oscillation frequencies suggest that the right hemisphere is more likely to generate the alpha rhythm. The segment was divided into six parts (see a–f in Figure 3C). The first cycle (designated with the numbers from 1 to 4 in Figure 3A) started with the minimum EEG amplitude at O2 (marked as a blue spot) and ended with the maximum amplitude at O1 (marked as a red spot). The next cycle (marked with the numbers from 5 to 7) ended with the maximal amplitude at the second generator, localized at O2. The synchronization of both sources subsequently occurred (at points 8 and 9). Of note, this system is not stable due to slowing of activity in the left hemisphere, which is clearly visible in the amplitude changes marked with the numbers from 16 to 21 (cf. Figure 3B). The activity at O2 (at point 16, 18, and 20) occurs before activity at O1 (at point 17, 19, and 21), suggesting that the source located at this electrode acts as a driver of the process. This was verified by analyzing the connectivity pattern using the DTF (data not shown).

### The importance of appropriate choice of reference electrode during EEG recording: existence of alpha rhythm generators outside the occipital lobe

**Figure 4:**
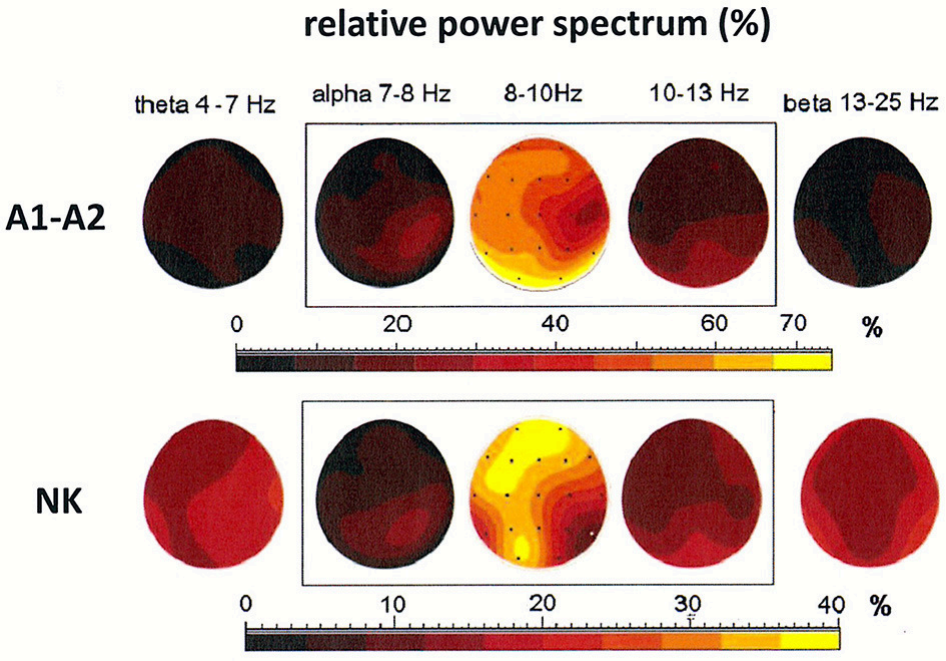
Relative power spectrum calculated for a 2-s segment of the EEG record in the third patient (54-year-old woman), recorded using two reference electrodes: linked earlobes (A1–A2) and neck (NK). The relative power spectrum is presented in five frequency bands (theta: 4–7 Hz; alpha: 7–8, 8–10, 10–13 Hz; beta: 13–25 Hz).

Both cases discussed above illustrate the existence of generators localized in posterior areas, particularly at derivations O1 and O2. Alpha rhythms can be also generated in other parts of the brain. The coexistence of several generators was demonstrated in the third patient, a 54-year-old woman (see Figure 4). The localization of generators depends on the choice of the reference electrode. The EEG signal was first registered to a reference electrode placed on the neck. This registration showed a maximum of the relative power spectrum of alpha band (8–10 Hz) in frontal and left occipital regions of the brain (see Figure 4). The same signal was then re-referenced to the linked earlobes reference (A1–A2). Here, the maximum of the relative power spectrum was localized to the posterior part of brain in left and right hemispheres, which was accompanied by a decrease of the relative power spectrum in frontal cortex. Next, the DTF was calculated to localize the generators and identify the directionality of EEG activity propagation and DTF strength (c.f. **Figures 6A,B**). The DTF was presented in each 1 Hz-frequency interval in the range of alpha bands. The dominance of the generator at O1 for both montages was verified (see graphs in **Figures 6A,B**). In addition, other generators were identified when the reference was placed on the neck, with one at P8 (with maximum strength at 9–10 Hz) and another at O2 (with maximum strength at 11–12 Hz). These DTF results changed dramatically when the signal was re-referenced to the linked earlobes (cf. **Figure 6B**). A second generator was identified at electrode Pz, which dominated for frequency of 10 Hz. Therefore, our data show that generators do not necessarily need to be localized in occipital or frontal parts of the brain, as was predicted by the Robinson et al. model (Robinson et al., 2001, 2003).

### Effect of data re-referencing (AVERAGE and REST)

**Figure 5:**
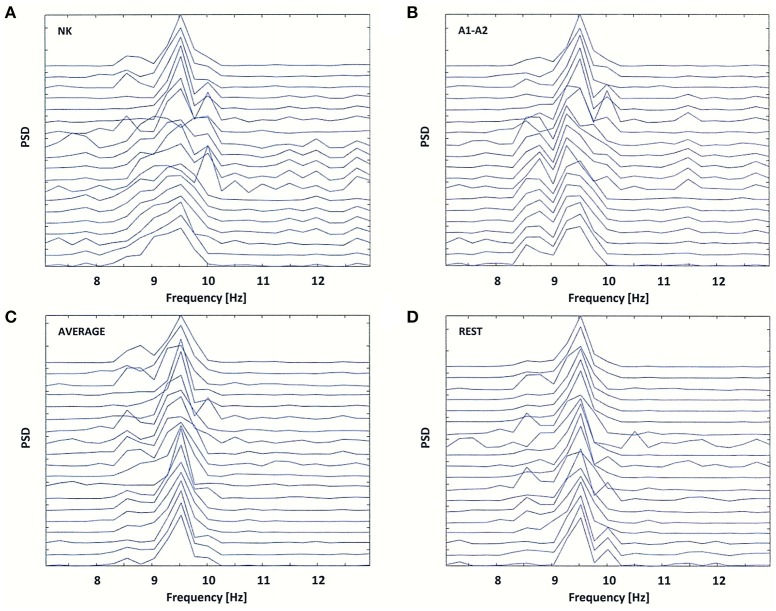
Power Spectral Density (PSD) for the same EEG segment as in Figure 4 **(A)**, and for the re-referenced data to: **(B)** linked earlobes reference (A1–A2), **(C)** average reference electrode (AVERAGE), and **(D)** reference electrode standardization techniques (REST).

**Figure 6:**
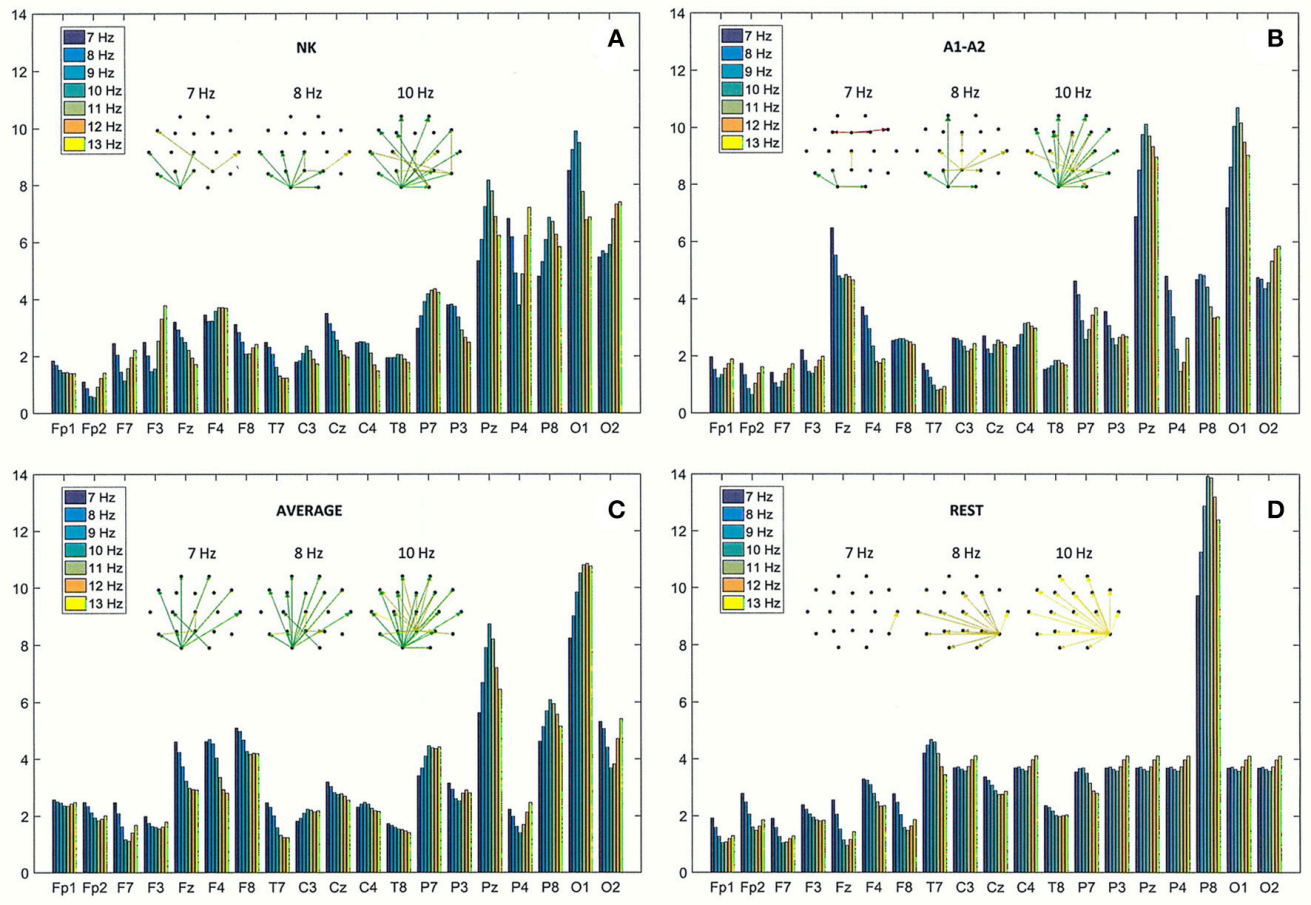
Directed Transfer Function (DTF) strength of outward links for the same EEG segment as in Figure 4 **(A)**, and for the re-referenced data to: **(B)** linked earlobes reference (A1–A2), **(C)** average reference electrode (AVERAGE), and **(D)** reference electrode standardization techniques (REST). Each chart contains graphs that represent the strongest 60% of connections between EEG channels, determined by the magnitudes and directions of the DTF calculated for three frequencies (7, 8, and 10 Hz).

The effect of data re-referencing is illustrated in Figures 5, 6. The power spectral density (PSD) of the 4-s segment of original EEG recorded in the third patient (54-year-old woman) is shown separately for each of four reference electrodes (NK, A1-A2, AVERAGE, and REST) in Figure 5. The frequency of split alpha peaks and the spatial distribution over the scalp surface are influenced strongly by choice of reference electrode.

The original data were re-referenced from the neck (NK) to the linked earlobes (A1–A2). The data referenced to the linked earlobes revealed two split alpha patterns: one in the occipital-parietal regions of brain and the second in frontal-central regions of brain. These patterns demonstrated a common dominant frequency of 9.5 Hz, but differed in the frequency of the second peak (8.5 Hz in the frontal-central lobe and 10 Hz in the occipital-parietal lobe; see Figures 5A,B).

The application of other reference electrodes (AVERAGE and REST) resulted in the reduction of low-frequency peak in frontal channels, and a sharpening of the peak at 9.5 Hz frequency (see Figures 5C,D). For the REST, the low-frequency alpha peak at the left and right temporal-parietal derivations (T7, T8, P7, P8) was appeared (see Figure 5D). Thus, it is likely that split alpha may result from an interaction between the occipital and temporo-parietal areas, rather than between left and right occipital hemispheres. However, this hypothesis can only be verified by applying REST to a high-density EEG dataset.

The strength of outward connections calculated from the adjacency matrices of DTF is presented for each of the four reference electrodes in Figure 6. Re-referencing the original EEG data to the linked earlobes reference (A1–A2) caused a reduction in strength at right parieto-occipital derivations (P4, P8, and O2), and an increase in strength at the central derivations (see Fz and Pz in Figures 6A,B). The results for the average reference also highlight the role of right frontal and parietal derivations (see F4, F8, and P8 in Figure 6C). The most outstanding results were obtained using the REST (cf. Figure 6D). The strength at electrode P8 was much higher than strength observed at other derivations. Interestingly, strength at occipital derivations was significantly reduced, reaching levels in both hemispheres that were comparable to levels observed in other channels in posterior brain regions.

The spatial distribution of the strongest 60% of connections between EEG channels are presented in graphs in Figure 6, for three frequencies (7, 8, and 10 Hz).

### The impact of volume conduction on the identification of split alpha

**Figure 7:**
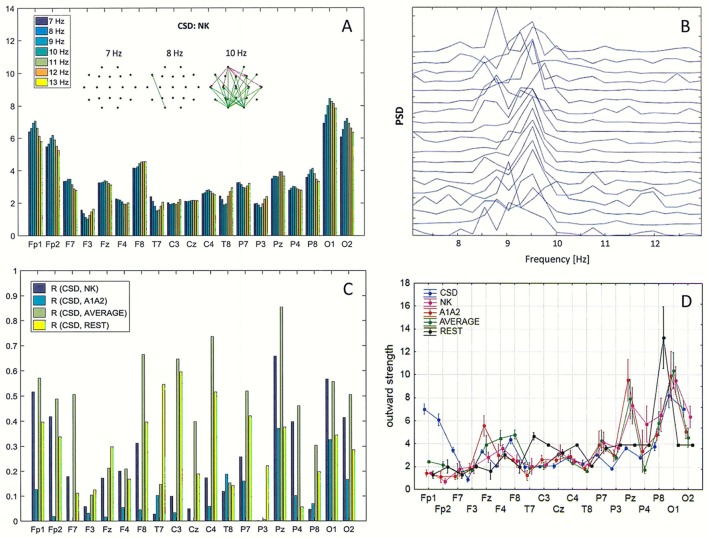
Impact of volume conduction on the identification of split alpha. **(A)** Directed Transfer Function (DTF) strength of outward links for the transformed data using the CSD transformation of the original EEG with the reference electrode placed at NK. Graphs represent the strongest 60% of connections between EEG channels, determined by the magnitudes and directions of the DTF calculated for three frequencies (7, 8, and 10 Hz). **(B)** Power Spectral Density (PSD) for the same data as in **(A)**. **(C)** Pearson correlation coefficients between CSD and four reference electrodes (NK, A1A2, AVERAGE, REST) at every EEG derivation. **(D)** Comparison of the average strength of outward links calculated for the transformed data using the CSD transform and for each of the four reference electrodes (NK, A1–A2, AVERAGE, REST).

The impact of volume conduction on the identification of split alpha is illustrated in Figure 7. The PSD of transformed data using the CSD transformation of the original EEG with the reference electrode placed at NK is showed in Figure 7B. The characteristic pattern of split alpha was completely abolished by the application of the CSD transform. In addition, although a few peak frequencies were still visible, their spatial distribution became disjointed. Comparing the PSD of the original EEG (see Figure 5A) with that of the transformed data (cf. Figure 7B), there was a shift in the dominant frequency of 9.5 Hz from the occipital lobe (the last seven channels: P7, P3, Pz, P4, P8, O1, O2) to the frontal lobe. In addition, the high-frequency alpha (10 Hz) was replaced by the low-frequency alpha (8.5–9 Hz).

Pearson correlation coefficients (R) between the PSD calculated for the CSD and the PSD for the four reference electrodes (NK, A1-A2, AVERAGE, REST) are presented in Figure 7C. The highest R values were between CSD and AVERAGE at almost all EEG derivations, and only at a few electrode locations did REST correlated with the CSD better than the AVERAGE (F3, Fz, T7, P3).

Figure 7D shows the average strength of outward links calculated with the transformed data using the CSD transform and with the four reference electrodes. These graphs were created by averaging the strength of outward links over all frequencies in the alpha band (from 7 to 13 Hz) shown in Figure 7A. Evaluating the results presented in Figure 7, reduction of volume conduction by applying the CSD transform resulted in an increase in strength of connections, particularly at the frontal derivations (Fp1, Fp2, F7), and a decrease in strength at central posterior derivation (Pz). Statistically significant differences (*p* < 0.05) between the CSD and each of the four reference electrodes were found for almost all EEG derivations, excluding C4, P4, and O1 for NK, T7, and C3 for A1–A2, T7, C3, O1, and O2 for AVERAGE.

## Discussion

Careful examination of the spatiotemporal maps of the EEG recordings, together with spectral analysis and analysis of connectivity using DTF, allowed for better explanation of the mechanism of split alpha effect generation. The SPD analysis allowed for the identification of at least two peaks with close frequencies in the alpha frequency range, consistent with the so called “split alpha” effect. The impact of window size on split alpha identification was tested, and a window size of 2 s was found to be optimal for this purpose. Next, the localization of split alpha peaks was examined using maps of the relative power spectra. These maps allowed us to compare the spatial distribution of power spectra in separate frequency bands (theta: 4–7 Hz; alpha: 7–8, 8–10, 10–13 Hz; beta: 13–25 Hz). We also calculated DTF, which allowed us to localize the generators and identify the directionality of EEG activity propagation. The index strength was calculated at every EEG channel to examine the importance of individual nodes in the network. Finally, spatiotemporal analysis of EEG amplitude evidenced several sources of alpha waves that underwent a dynamical process. One of the hemispheres was more likely to generate an alpha rhythm, which may be due to the slightly different oscillation frequencies of the two interconnected generators (i.e., O1 and O2).

Robinson et al. predicted the split alpha effect by analyzing a modified model of alpha rhythm generator (Robinson et al., 2001, 2003). They concluded that the frequency difference between two generators localized in frontal and occipital lobes could result in overlapping double peaks. In this study, we demonstrated that split alpha can be generated from the interaction between at least two distant alpha generators located in different brain regions that are not necessarily in occipital or frontal lobes. Moreover, the mechanism of split alpha generation may differ by individual, and hemispheric and fronto-posterior asymmetry may impact this variability. A decrease in cortico-cortical and cortico-thalamic propagation delay may also contribute to the frequency shift.

In this study, we evaluated, for the first time, the impact of reference electrode placement on the split alpha effect. Our data demonstrate the importance of reference electrode placement at the level of signal recording. Indeed, our results suggest that the monopolar montage with a reference electrode placed in an appropriate head position allows for better localization of alpha waves generators. Results obtained with the original data were also compared with results of re-referenced data, using the average reference electrode (AVERAGE) (Nunez and Srinivasan, 2006) and reference electrode standardization techniques (REST) (Yao, 2001, 2017; Zhai and Yao, 2004; Yao et al., 2005). The AVERAGE and REST techniques may be more appropriate, however, for application in high-density EEG recordings, which yield low re-referencing reconstruction errors (Liu et al., 2015). For low-density EEG recordings (e.g., 21-channel montage), the average global relative error of AVERAGE and REST from a three-layer spherical model was estimated to be ~21 and ~13%, respectively (Liu et al., 2015). However, in clinical practice the standard 10–20 system with 19 electrodes is more frequently used to evaluate EEG data in ambulatory patients. In such circumstances, more conventional references such as linked earlobes (A1–A2), neck (NK), or chin (S1), are still useful. It is important to note, however, which reference is used in each specific case. As a general rule, the reference electrode should be placed as far as possible from the source of brain activity. Further work is needed to better understand the mechanisms of split alpha generation using high-density EEG data with AVERAGE and REST techniques. Future work should also evaluate the impact of age on mechanisms of split alpha generation, particularly in healthy subjects who range widely in age.

Of note, the problem of volume conduction should be considered when the DTF method is applied (Kaminski and and Blinowska, 2017). CSD (Kayser and Tenke, 2006a,b, 2015; Kayser, 2009) may be one solution. However, also in this case a high-density EEG data should be analyzed.

## Conclusions

We found that the split alpha peak was a common phenomenon observed in EEG data. However, identification of this phenomenon depended on several methodological choices.

For occipital alpha wave generators, the presence of occipital split alpha peaks may be associated with variation in interhemispheic connectivity, which leads to relatively independent activity of occipital alpha wave generators in left and right hemispheres.

The example re-referenced data using the REST technique suggested that the split alpha effect may be driven by an interaction between the occipital and temporo-parietal areas, rather than between left and right occipital lobes. This hypothesis should be confirmed by applying the REST technique to high-density EEG data.

CSD is frequently used to reduce the effect of volume conduction. Another feature of the CSD transform is it is free from reference effects, so the highest correlation between CSD and REST should be expected. However, our data showed that CSD correlated better with AVERAGE than with REST, which may be due to the use of low-density EEG data.

In sum, our data suggest that recording montage, duration of the analytical window, and EEG activity dynamics should be considered when collecting and analyzing EEG data. Our results demonstrate an association between the composition of the SPD within alpha wave frequencies and connectivity patterns between different alpha rhythm generators.

## Ethics statement

This study was carried out in accordance with the recommendations of Ethics Committee of the Medical Centre for Postgraduate Education in Warsaw, Poland with written informed consent from all subjects.

## Author contributions

EO: Conception of the work, EEG analysis, wrote the manuscript; PB: Conception of the work, acquisition of EEG data; wrote the manuscript; AS: Conception of the work, interpretation of EEG data, wrote the manuscript.

### Conflict of interest statement

The authors declare that the research was conducted in the absence of any commercial or financial relationships that could be construed as a potential conflict of interest.
